# TRPML2 is an osmo/mechanosensitive cation channel in endolysosomal organelles

**DOI:** 10.1126/sciadv.abb5064

**Published:** 2020-11-11

**Authors:** Cheng-Chang Chen, Einar Krogsaeter, Elisabeth S. Butz, Yanfen Li, Rosa Puertollano, Christian Wahl-Schott, Martin Biel, Christian Grimm

**Affiliations:** 1 Department of Pharmacy–Center for Drug Research, Ludwig-Maximilians-Universität, Munich, Germany.; 2 Walther Straub Institute of Pharmacology and Toxicology Faculty of Medicine, Ludwig-Maximilians-Universität, Munich, Germany.; 3 Center for Genomic Medicine, Department of Neurology, Massachusetts General Hospital, Harvard Medical School, Boston, MA, United States.; 4 Cell and Developmental Biology Center, National Heart, Lung, and Blood Institute, National Institutes of Health, Bethesda, MD, United States.; 5 Institute for Neurophysiology, Hannover Medical School, Hannover, Germany.

## Abstract

Endolysosomes are dynamic, intracellular compartments, regulating their surface-to-volume ratios to counteract membrane swelling or shrinkage caused by osmotic challenges upon tubulation and vesiculation events. While osmosensitivity has been extensively described on the plasma membrane, the mechanisms underlying endolysosomal surface-to-volume ratio changes and identities of involved ion channels remain elusive. Endolysosomes mediate endocytosis, exocytosis, cargo transport, and sorting of material for recycling or degradation. We demonstrate the endolysosomal cation channel TRPML2 to be hypotonicity/mechanosensitive, a feature crucial to its involvement in fast-recycling processes of immune cells. We demonstrate that the phosphoinositide binding pocket is required for TRPML2 hypotonicity-sensitivity, as substitution of L314 completely abrogates hypotonicity-sensitivity. Last, the hypotonicity-insensitive TRPML2 mutant L314R slows down the fast recycling pathway, corroborating the functional importance of hypotonicity-sensitive TRPML2. Our results highlight TRPML2 as an accelerator of endolysosomal trafficking by virtue of its hypotonicity-sensitivity, with implications in immune cell surveillance and viral trafficking.

## Teaser

Protein trafficking is mediated by tubulation/vesiculation of endosomes and is regulated by osmo/mechanosensitive TRPML2.

## INTRODUCTION

The endolysosomal system consists of early endosomes, late endosomes (LE), lysosomes (LY), and recycling endosomes (RE) alongside autolysosomes (AL) and phagosomes. These are quasi-spherical organelles that play essential roles in a range of physiological processes. Intracellular cargo such as receptors trafficked for recycling or degradation or inflammatory mediators such as cytokines and chemokines for immediate release are transported via endolysosomal trafficking routes. Dysfunction of endolysosomal trafficking and cargo sorting appear broadly pathogenic, manifesting in lysosomal storage disease, metabolic disorders, and infectious diseases (*1*–*4*). Intracellular compartmental trafficking is mediated by a series of tubulation and vesiculation events, dynamically altering endolysosomal surface-to-volume ratios and reducing radii of curvature (*5*). According to the Young-Laplace equation for spheres (vesiculation) ∆*p* = 2γ/*R* and for cylinders (tubulation) ∆*p* = γ/*R*, the pressure difference (∆*p*) between the luminal side and the cytosolic side of the endolysosomal surface increases when the radii (*R*) of the spheres or cylinders decrease. Accordingly, an increase in ∆*p* leads to an increase in surface tension (γ). Membrane tension may not only be generated by increasing surface-to-volume ratio during vesiculation but could also result from spontaneous bending of the lipid bilayer during tubulation (*6*–*7*).

On the cell surface, several volume-regulated and osmotically sensitive ion channels have been identified and characterized, such as TRPV4 and LRRC8 (*8*, *9*). These ion channels sense surface-to-volume ratio variations and correspondingly mediate anion and cation fluxes across the plasma membrane, regulating the cytosolic ion concentration and osmolyte-driven osmosis. Compared to whole cells, endolysosomes are much smaller, and thus, variations of organellar surface-to-volume ratios are very rapid and severe. While whole-cell diameters range from 10 to 20 μm, the diameter of spherical vacuoles ranges from 0.1 to 0.5 μm, while tubules can be much narrower (0.05 μm). However, osmolarity or surface-to-volume ratio-sensitive endolysosomal ion channels remain unidentified.

Several endolysosomal cation channel activities have been observed on endolysosomal membranes and have been suggested to play critical roles in organelle trafficking, fusion, and fission (*10*, *11*). The mucolipin [The transient receptor potential mucolipin (TRPML)] family and the two-pore channels belong to the transient receptor potential (TRP) superfamily and are often discussed in the context of Ca^2+^/Na^+^ transport and their ability to release cations from the endolysosomal lumen, mediating organellar fusion and fission events (*11*–*13*). They play essential roles in several physiological and pathological states, including lysosomal storage disorders, metabolic diseases, (cancer) cell migration, and metastasis formation, as well as infectious diseases (*2*, *14*–*17*). These cation channels are particularly highly expressed in a variety of quasi-spherical organelles, which may contribute to the regulation of the surface-to-volume ratio of the tubules and vesicles during fission and trafficking processes. Our recent study employed a novel selective TRPML2 channel agonist, ML2-SA1, to demonstrate that TRPML2 activation in the endolysosomal system promotes the release of the chemoattractant CCL2 from lipopolysaccharide (LPS)–activated macrophages and subsequent macrophage migration (*18*). Here, we further identified that TRPML2 acts as an osmo/mechanosensitive cation channel in endolysosomes derived from innate immune cells with a predominant activation in fast recycling, Rab4A-positive vesicles, enabling rapid adaptation to osmotic changes upon tubulation and vesiculation processes.

## RESULTS

### Characterization of hypotonicity-sensitive TRPML2 channels in endolysosomes

**Fig. 1:**
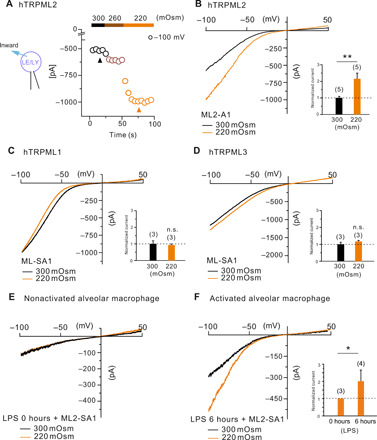
Hypotonic stimulation-evoked TRPML2 currents. Whole-LE/LY patch-clamp recordings. Inward current indicates cations flowing from the vesicle lumen into the cytosol (bath). (**A**) Time course of whole LE/LY recordings with ramp protocols (every 5 s; holding potential, 0 mV). Recordings at time points (indicated) were used for the current-voltage (*I*-*V*) relationships in (**B**). Representative hypotonic conditions increased ML2-SA1 (10 μM)–elicited currents from LE/LY isolated from hTRPML2 expressing HEK293 cells. (**C** and **D**) Representative osmolarity-insensitive inward currents recorded from hTRPML1- or hTRPML3-expressing HEK293 cells. (**E** and **F**) Representative hypotonicity-sensitive TRPML2-like currents recorded from isolated LE/LY from nonactivated (E) and LPS-activated (F) alveolar macrophages. Statistical summary of data, as shown as fold increase compared to the respective currents under physiological osmolarity, is shown as an inset of *I*-*V* plots. The current amplitudes at −100 mV were extracted from individual ramp current recordings. The numbers of individual organelles are in parentheses. Data are represented as mean ± SEM. *P < 0.05 and **P < 0.01; Student’s *t* test, unpaired. n.s., not significant difference.

To identify osmotically sensitive cation channels in the endolysosomal system, we used a whole-intracellular organelle patch-clamp approach to measure current fluxes across intact organelle membranes (*18*–*20*). Exposure to a hypotonic cytosolic solution (220 mOsm) markedly increased inwardly rectifying cation currents from endolysosomes isolated from TRPML2-transfected human embryonic kidney–293 (HEK293) cells. We initially used the LE/LY patch-clamp approach to identify hypotonicity-induced ML2-SA1–evoked currents in TRPML2-transfected HEK293 cells (Fig. 1, A and B). In contrast to TRPML2, neither the TRPML1 channel nor TRPML3 was found to be sensitive to hypotonic stimulation (Fig. 1, C and D). We further observed that pan-TRPML agonist (ML-SA1)–evoked TRPML2-like currents from human and mouse variants of TRPML2 were also increased under hypotonic conditions (fig. S1), as well as currents evoked by applying hypotonic solution alone (fig. S2). Recent studies demonstrated that TRPML2 expression and channel activity are significantly increased in endolysosomes isolated from LPS-stimulated macrophages (*18*, *21*). Endolysosomal recordings from primary alveolar macrophages (AMØ) illustrate that the endogenously expressed TRPML2 channel is also sensitive to hypotonic stimulation (Fig. 1, E and F). These data indicate that TRPML2 is a hypotonicity-sensitive cation channel in endolysosomes, both in an overexpression system and in endogenously expressing activated macrophages.

### Phosphoinositide binding pocket involved in hypotonicity-sensitivity of TRPML2

**Fig. 2:**
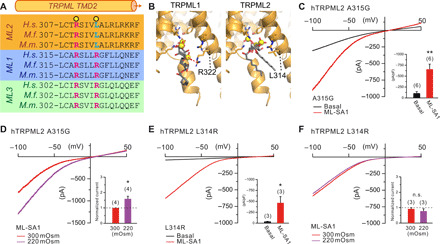
PIP_2_ binding pocket contributes to hypotonicity-sensitive TRPML2 activation. (**A**) Sequence alignment of the PIP_2_ binding pocket transmembrane domain 2 (TMD2) of TRPML1-3 as illustrated. To highlight conserved residues, amino acid sequences for *Homo sapiens* (*H.s.*), *Macaca fascicularis* (*M.f.*), and *Mus musculus* (*M.m.*) are shown. Yellow circles mark residues suggested to be essential for PI(3,5)P_2_ binding at TRPML1 based on the cryo-EM structure and therefore may be necessary for PIP-dependent surface-to-volume ratio sensitivity. Positive charges of PI(3,5)P_2_-interacting arginines are denoted by pink font color, while cyan marks hydrophilic leucines conserved and present in TRPML2 only. (**B**) Comparison of the TRPML2 homology model PIP binding pocket and that experimentally resolved for TRPML1. In TRPML1, the PI(3,5)P_2_ C1-phosphate can be seen to interact with R322, while the substitution of the arginine with a leucine in TRPML2 disfavors a similar interaction. (**C** to **F**) Whole-LE/LY recordings of ML-SA1 (10 μM) elicited TRPML2 currents under isotonic or hypotonic conditions from TRPML2 A315G (C and D) and TRPML2 L314R (E and F) expressing HEK293 cells. Data are represented as mean ± SEM. *P < 0.05, **P < 0.01, Student’s *t* test, paired.

The coupling of cellular osmolarity change to pore opening could be mediated by a mechanical stimulus and interactions of a membrane lipid with a binding pocket on the channel (*9*). The binding pockets and interacting mechanisms of phosphatidylinositol phosphates (PIPs) with TRPML1 and TRPML3 have been revealed by cryo–electron microscopy recently (*22*–*25*). Given the irresponsiveness of TRPML1 and TRPML3 to osmolarity changes and the possible involvement of phosphoinositides in connecting membrane tension with channel activity, we hypothesized that the unique osmolarity sensitivity of TRPML2 might be conferred by differences in the channel’s PI(3,5)P_2_binding pocket. Accordingly, we used the published TRPML1 and TRPML3 structures to map these (Fig. 2A). On the basis of the sequence alignment and homology modeling of the PI(3,5)P_2_binding pocket, we found TRPML2 to share several critical residues required for PI(3,5)P_2_ activity with TRPML1 and TRPML3 but lacking an arginine at position 314 (TRPML1 R323, TRPML2 L314, and TRPML3 R309; Fig. 2, A and B). The positive charge of arginines in the PIP pocket is likely of functional relevance, providing a charged interaction partner of the negatively charged phosphoinositide head. On the basis of the hypothesis that osmoregulation might be conferred by phosphoinositide recruitment, we reasoned that mutation of L314 into its positively charged TRPML1/3 counterpart would abolish TRPML2 osmoregulation. We subsequently analyzed mutant isoforms L314R and the neighboring, non-PIP–interacting A315G in whole-LE/LY patch-clamp experiments (Fig. 2, B to E). Both A315G and L314R remained responsive to ML-SA1 (Fig. 2, B and D) and ML2-SA1 and PI(3,5)P_2_ (fig. S3), indicating that the mutant channel remained functional. However, the TRPML2 L314R mutation was found to abolish the effect of hypotonic stress, while its adjacent A315G mutation remained able to sense hypotonic stimulations similar to wild-type (WT) TRPML2 (Fig. 2, C and E). These data suggest that the L314R mutation specifically impairs the channels’ responsiveness to changes in cytosolic osmolarity while it does not affect principal activation gating. While this experiment demonstrated that L314 within the phosphoinositide binding site is a crucial determinant of hypotonicity sensing in TRPML2, other channel domains very likely also contribute to this function. In agreement with this hypothesis, introducing leucine residues at positions corresponding to L314 in TRPML1 (R322L) or TRPML3 (R309L) was not sufficient to endow these channels with hypotonicity sensing (fig. S4).

### Mechanosensitivity of TRPML2 on endolysosomes

**Fig. 3:**
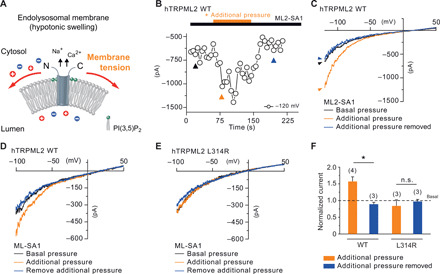
Mechanical force stimulus-evoked TRPML2 currents. (**A**) Hypotonic stimulation changes the osmotic gradient, which induces swelling and directly increases lateral tension in the lipid bilayer. The hypotonicity/mechanosensitive ion channel converts mechanical stimuli into ion flux. (**B** to **E**) Whole-LY/LE currents were recorded from hTRPML2 WT and L314R mutant isoform-expressing HEK293 cells ± pressure-induced membrane physical force. Time course (B) and representative traces (C) of pressure-enhanced ML2-SA1–evoked currents from TRPML2 WT channels. Additional pressure was directly applied to the membrane patch (+0.06 ml of a 1-ml syringe) and released after 1 min. (D and E) ML-SA1–evoked TRPML2 inward cation currents were increased by additional physical force on LE/LY from TRPML2 WT-expressing HEK293 cells (D) but not from L314R expressing HEK293 cells (E). (**F**) Statistical summary of date as shown as fold increase compared to the respective currents without additional pressure. The numbers of individual organelles are in parentheses. Data are represented as mean ± SEM. *P < 0.05, one-way ANOVA, repeated measures, followed by Bonferroni post hoc test.

Most mechanosensitive ion channels are regulated by osmotic stress, such as TRP vallinoid (TRPV) and canonical (TRPC) family channels (*26*). To assess whether physical forces mediate TRPML2 activation through affecting the lipid membrane tension, we applied additional pressure (+0.06 ml of a 1-ml syringe; ~+40 mm Hg) into the lumen of the isolated whole organelles with the patch pipette, mimicking the expected increased membrane tension upon hypotonic stimulation. The additional positive pressure increased mechanical membrane tension and, correspondingly, TRPML2 inward cation current amplitudes (Fig. 3). In contrast, the L314R mutant channel did lose not only the sensitivity of hypotonic stimulation but also the mechanosensitivity (Fig. 3, E and F). These data suggest that TRPML2 is activated by hypotonic stimulation and also by mechanical forces in membrane patches.

### TRPML2 in recycling endosomes is sensitive to osmolarity changes

As reported recently, TRPML2 promotes trafficking and secretion of the chemokine CCL2 from murine macrophages via the apical endosomal pathway (*18*). To demonstrate the osmosensitivity of TRPML2 in apical endosomes, we cotransfected transferrin receptor (TfR) with TRPML2 and patch-clamped TfR^+^ and TRPML2^+^ RE. Similarly, as demonstrated for LE/LY, TRPML2 currents were increased in TfR^+^ RE under hypotonic conditions (fig. S5, A and B). Transferrin (Tf)–loaded RE, isolated from LPS-stimulated alveolar macrophages, likewise showed an increase of TRPML2 channel activity under hypotonic conditions (fig. S5C).

### Substantial TRPML2 activities in fast recycling endosomes

**Fig. 4:**
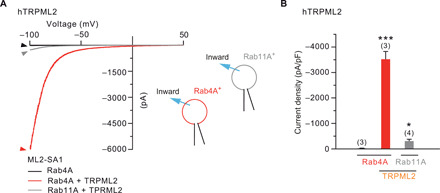
Whole fast/slow recycling endosome recordings. (**A**) Representative hTRPML2 currents recorded from Rab4A-positive (red) or Rab11A-positive (gray) RE isolated from TRPML2-coexpressing HEK293 cells. (**B**) Statistical summary of data, as shown in A. Numbers of individual organelles are in parentheses. Data are represented as mean ± SEM. *P < 0.05 and ***P < 0.001; one-way ANOVA, repeated measures, followed by Bonferroni post hoc test.

We have previously demonstrated that pharmacological TRPML2 activation with ML2-SA1 resulted in a significant enhancement of Tf trafficking and recycling through the apical endosomal system within the first ~5 min, corresponding to the fast recycling pathway (*18*). Rab4A and Rab11A are used to distinguish fast and slow recycling endosomes, with Rab4A mediating rapid recycling and Rab11A mediating slow recycling activities (*27*). We cotransfected TRPML2 with either Rab4A or Rab11A and measured the respective TRPML2 activities. These measurements revealed that TRPML2 channel activities were markedly higher in Rab4A-positive fast RE compared to Rab11A-positive slow RE (Fig. 4), suggesting that TRPML2 is highly active in the fast RE pathway.

### The hypotonicity-insensitive TRPML2 mutant isoform L314R slows down the fast recycling pathway

**Fig. 5:**
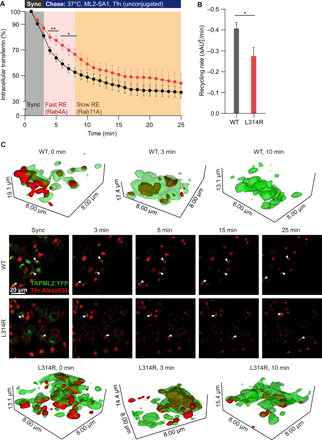
Cells expressing the hypotonicity/membrane lateral tension-insensitive TRPML2 variant L314R exhibited slower Tf recycling through the fast recycling pathway. Tf trafficking experiments were performed on WT and L314R TRPML2-overexpressing HEK293 cells. (**A**) Plot of intracellular Tf normalized to initially loaded Tf over time, images taken at 1-min intervals. Significantly more Tf remained inside the L314R-mutant cells within the first 5 min of chasing compared to WT-transfected counterparts. (**B**) Calculation of the fast recycling rate (slope for the initial 5 min of recycling) showed the L314R mutant to possess a significantly slower fast recycling rate. (**C**) Two-dimensional images from representative images for data presented in (A) and (B). Arrowheads indicate Tf-positive endosomes expressing TRPML2. The average of three independent experiments is shown. Three-dimensional panels provide higher resolution of Tf (red)–containing endosomes expressing WT or L314R TRPML2 (green) at the indicated time points of chasing (0, 3, and 10 min). Corresponding videos are provided (movies S1 to S6). *P < 0.05, **P < 0.01, two-way ANOVA, followed by Bonferroni post hoc test for (A) and *t* test for (B).

To validate the effect of hypotonicity/mechanosensitivity of TRPML2 in recycling processes, hTRPML2 WT and the hypotonicity/lateral membrane tension-insensitive isoform (L314R) were expressed in HEK293 cells. The cells were pulsed with fluorescently labeled Tf and chased in the presence of unlabeled Tf and the TRPML2 agonist ML2-SA1 (30 μM). The obtained data imply that the loss-of-function (LOF) mutant isoform (hTRPML2 L314R) exhibited a significantly slower Tf recycling rate within the fast recycling time frame (initial 5 min) compared to WT TRPML2 (Fig. 5). Hypothesizing that TRPML2 might accelerate tubulation and vesiculation events in Tf-containing endosomes, we assessed the localization of TRPML2. We found TRPML2 to localize to tubules in both overexpressing HEK293 cells and endogenously expressing macrophages (Fig. 5C, fig. S6, and movies S1 to S6). Furthermore, TRPML2-expressing tubular endosomes could be loaded with fluorescent Tf, which were emptied during the chase period (Fig. 5C). Together, our findings show the hypotonicity/membrane lateral tension sensitivity of TRPML2 to be crucial for appropriately regulating TRPML2 activity within the Rab4A fast recycling pathway.

## DISCUSSION

In this study, we report that TRPML2 is a hypotonicity-sensitive and mechanosensitive cation channel in intracellular organelles, isolated from either overexpressing HEK293 cells or endogenously expressing activated macrophages. Until now, osmosensitive and mechanosensitive ion channels had not been identified in intracellular compartments. We applied the whole-endolysosome patch-clamp technique to characterize TRPML2 as the only hypotonicity-sensitive ion channel of the TRPML subfamily in the endolysosomal system. Using structure-guided site-directed mutagenesis, we found the TRPML1/TRPML3-like point mutation L314R within the TRPML2 PI(3,5)P_2_ binding pocket to abrogate TRPML2 osmo/mechanosensitivity. We mutated TRPML1 and TRPML3 at the positions equivalent to L314 in TRPML2, but this did not result in gain of osmosensitive function. Together, our results imply that TRPML2 osmo/mechanosensitivity requires L314, while TRPML1 and TRPML3 require more than a single amino acid substitution to respond to osmotic/mechanical force.

Recent studies have suggested that TRPML2 promotes recycling in innate immune cells (*18*, *21*). Fast recycling of endolysosomal compartments is accompanied by active tubulation, vesiculation, and rapid changes of surface-to-volume ratios. These dynamic changes increase membrane tension in the RE membrane surface during the fast recycling process. Here, we observed that TRPML2 colocalizes with Tf in tubular structures and observe substantial TRPML2 channel activity in Rab4A-positive fast RE compared with Rab11A-positive slow RE. We furthermore observed the LOF mutant L314R to slow down the fast recycling pathway compared to its WT counterpart, likely resulting from high TRPML2 channel activity in fast RE. These findings suggest that osmo/mechanosensitive TRPML2 plays a role in fast recycling pathways, enabling rapid recycling through enhancement of the adaptation processes following the surface-to-volume-ratio-change of tubulation and vesiculation.

On the basis of the studies of hypotonicity-sensitive ion channels in the plasma membrane, hypotonicity-sensitive mammalian TRP channels often sense mechanical stress, such as TRPV2, TRPC1, TRPP2, and TRPM3. Our whole-endolysosomal patch-clamp experiments show that both ~+40 mm Hg and 220 mOsm stimuli further enhance TRPML2 ion flux, the stimuli being close to the typical range of pressure and osmotic challenge for osmo/mechanosensitive TRP channels on the plasma membrane (*28*). Multiple mechanisms of osmo/mechanosensitivity are suggested for plasma membrane TRP channels. The first hypothesis proposes that hypotonic stimuli lead to swelling and increase in membrane tension, causing the membrane lipids to pull away from the channel protein leading to a conformational change of the channel. The second hypothesis suggests that accessory ligands between lipids and channel proteins transduce a response to mechanical forces on the membrane. The third model is an indirect pathway, suggesting that the channel is not directly sensing mechanical forces via lipid-protein interactions.

In contrast, sometimes, mechanical forces result in secondary signaling components (lipases, kinases, and heterotrimeric GTP-binding proteins) to facilitate channel activation pathways such as for TRPV4 (*29*). Mutagenesis experiments suggest that the PI(3,5)P_2_ binding pocket impacts the mechanism of osmo/mechanosensitivity. However, the scenario underlying the osmo/mechanosensitivity of TRPML2 requires high-speed adaptation pressure (1 mmHg within 10 ms) by endolysosomal patch clamp, which allows precise observation of size changes of the organellar compartment. It remains unlikely that the TRPML2 volume-to-surface ratio sensitivity is mediated by second messengers, which require more time to exert an effect downstream of their activation. The identification of a single amino acid crucial for osmo/mechanosensitivity further supports this conclusion.

On the other hand, endolysosomes are dynamic, quasi-spherical organelles in the absence of basic support by the cytoskeleton and filaments. The frequency and rate of variation of size and membrane tension considerably exceed the fluctuations observed at the plasma membrane. Endolysosomal ion channels regulate osmolyte influx and efflux across the organellar membrane and promote surface-to-volume adaptation (*5*). These changes may require a more massive and faster reaction in fast endosomal recycling compartments that rely on sustained rapid trafficking, particularly in the scavenging, surveilling, and signaling macrophages of the innate immune response. These results suggest that the osmo/mechanosensitive TRPML2 may play an essential role in fast recycling and secretion processes of activated innate immune cells.

A growing number of studies imply essential roles for TRPML2 in a number of physiological and pathophysiological processes including innate immune response, adaptive immune response, tumor progression (e.g., glioma), and virus infection (*30*–*32*). Our work reveals a novel and exciting role for TRPML2 in the response to osmo/mechanical stimuli as a way to enhance specific trafficking pathways in macrophages. It may also imply that the surface-to-volume-ratio changes of tubulation and vesiculation of endolysosomes during immune surveillance is necessary to maintain physiological functions in immune cells. In summary, TRPML2 is the first characterized osmo/mechanosensitive endolysosomal ion channel on intracellular membranes, making this channel unique among the other endolysosomal cation channels, particularly its relatives TRPML1 and TRPML3.

## MATERIALS AND METHODS

### Endolysosomal patch-clamp experiments

Whole-endolysosome recordings have been described previously in detail (*18*–*20*). In brief, for whole-LE/LY patch-clamp recordings, isolated intact vesicles from cells were manually isolated after YM201636 treatment (HEK293 cells, 1 μM o/n; macrophages, 800 nM, 1 hour). For whole-RE patch-clamp recordings, HEK293 cells were transfected with the markers Rab4A-mCherry (for fast-RE) or Rab11A-DsRed (for slow-RE) or TfR-mCherry (for RE), respectively, and treated with 1 μM vacuolin overnight. Human TRPML2 WT, mouse TRPML2 WT, and point mutation isoforms of human TRPML2 [C-terminally fused to yellow fluorescent protein (YFP)] were transiently transfected into HEK293 cells using TurboFect Transfection Reagent (Thermo Fisher Scientific). Preparation of primary alveolar macrophages has been described previously (*18*). Animals were used under approved animal protocols and the University of Munich (LMU) Institutional Animal Care Guidelines. Cells were treated with compounds at 37°C and 5% CO_2_. YM201636 was obtained from Chemdea (CD0181), and vacuolin from Santa Cruz Biotechnology (sc-216045). Compounds were washed out before patch-clamp experimentation. Alveolar macrophages were used for experiments within 2 to 10 days after isolation. Currents were recorded using an Axonpatch 200B (Molecular Devices) and pClamp v10 software (Molecular Devices). Data were digitized at 40 kHz and filtered at 2.8 kHz. Capacitive transients were canceled by the compensation circuit of the Axonpatch 200B amplifier.

Recording glass pipettes were polished and had a resistance of 4 to 8 megaohm. Liquid junction potential was corrected. For the application of PI(3,5)P_2_ (AG Scientific) or small-molecule agonists (ML2-SA1 and ML-SA1), the perfusion system was applied to exchange cytoplasmic solution by cytoplasmic solution containing agonist completely. Unless otherwise stated, the cytoplasmic solution (bath) contained 140 mM K-MSA (methanesulfonate), 5 mM KOH, 4 mM NaCl, 0.39 mM CaCl_2_, 1 mM EGTA, and 10 mM Hepes (pH was adjusted with KOH to 7.2). Luminal solution (pipette) contained 140 mM Na-MSA, 5 mM K-MSA, 2 mM CaMSA, 1 mM CaCl_2_, 5 mM Hepes, and 5 mM MES (2-(N-Morpholino)-ethane sulfonic acid) (pH was adjusted with NaOH to 7.2). For optimal recordings of TRPML1, luminal pH was adjusted to 4.6, and Na-MSA was used in the luminal solution. For optimal recordings of TRPML2, luminal pH was adjusted to 7.2, and Na-MSA was used in the luminal solution. For optimal recordings of TRPML3, luminal pH was adjusted to 7.2 and K-MSA. All statistical analysis was done using Origin software.

### Tf trafficking assay

HEK293 cells were split on 18-mm glass coverslips coated with poly-l-lysine. Cells were transfected with constructs of interest [hTRPML2(WT):YFP and hTRPML2(L314R):YFP] using TurboFect Transfection Reagent (Thermo Fisher Scientific), according to the manufacturer’s instructions. Transfections were left for 18 hours before the experiment.

Upon starting the live-cell Tf trafficking assay, cells were first washed once with phosphate-buffered saline (PBS) (Thermo Fisher Scientific) and starved in serum-free Dulbecco’s modified Eagle’s medium (DMEM) (Thermo Fisher Scientific) for 30 min at 37°C. Subsequently, cells were washed with PBS and synchronized by incubation in serum-free DMEM for 10 min at 4°C. Cells were washed once more with ice-cold PBS and pulsed with fluorescently labeled Tf-Alexa555 (20 μg/ml) in serum-free medium for 30 min at 37°C. Following the pulse, cells were washed twice with ice-cold PBS, synchronization medium for imaging was added (ice-cold phenol red–free DMEM with Hepes, Thermo Fisher Scientific) and immediately transferred to a Zeiss LSM880 confocal microscope for recording, preequilibrated at 37°C in a temperature-controlled climate chamber. Images were taken using a 63×/1.20 water-based objective/photomultiplier tube detector and a resolution of 1024 × 1024 pixels (pixel size 0.22 μm) to compromise between endosomal (spatial) and temporal resolution. The initial image was taken using the green (488 nm) and red (561 nm) channels for TRPML2:YFP and loaded Tf, respectively. Subsequently, the time lapse acquisition was started, capturing Tf intensities (562 nm) every minute over a 25-min time frame. At 3 min of imaging, the chase was commenced upon adding 37°C medium containing ML2-SA1 (30 μM) and a high-dose unconjugated Tf (200 μg/ml). The analysis was performed using the MeanROI function of ZenBlue, selecting cells expressing TRPML2:YFP for analysis. Loss of fluorescent Tf within the 5 min following the chase (i.e., between 3 and 8 min) was attributed to fast recycling activity, and fast recycling kinetics were calculated on the basis of this time window. Statistical analysis was performed using GraphPad Prism 8. Time lapse plots were analyzed as a repeated-measures analysis of variance (ANOVA) followed post hoc by a Bonferroni test, while recycling rates were analyzed by a Student’s *t* test.

For high-resolution images of Tf-loaded tubules, we opted to fix the cells to avoid endosomal movement during image acquisition, which could underlie false-positive identification of endosomal tubules. The experiment was performed as described above, until the addition of synchronization medium. Instead, the cells were either immediately fixed or chased for 10 min [DMEM +30 μM ML2-SA1 and unconjugated Tf (200 mg/ml)]. Fixation was performed using 4% paraformaldehyde (PFA) at room temperature. The cells were rinsed with PBS and mounted on microscope slides. Images were acquired using a Zeiss LSM880 confocal microscope, using a 100×/1.46 oil-based objective, acquiring *z* stacks at 0.34-μm intervals covering the TRPML2-fluorescent area. *Z* stacks were deconvolved using the Fiji plugins “Diffraction PSF 3D” and “Iterative Deconvolve 3D,” and regions of interest cropped to 8 μm by 8 μm. Three-dimensional (3D) surfaces were visualized using the Fiji plugin “3D Viewer,” applying uniform thresholds, and setting TRPML2 transparency to 0.5.

### Immunofluorescence

Preparation of primary murine bone marrow–derived macrophages (BMDMØ) has been described previously (*18*). Animals were used under approved animal protocols and the University of Munich (LMU) Institutional Animal Care Guidelines. BMDMØ were stimulated using LPS (1 μg/ml) and fixed using 2% PFA at room temperature for 30 min. Cells were blocked for 90 min at room temperature (0.5% Triton X-100 and 5% normal goat serum in PBS). The staining solution was prepared as PBS with 0.5% Triton X-100 and 1% normal goat serum. Primary antibody incubation was performed overnight at 4°C using a rabbit primary anti-TRPML2 antibody (gift from R. Puertollano; 1:100 in staining solution). Coverslips were rinsed three times and stained with secondary anti-rabbit Alexa488 for 2 hours at room temperature (1:500 in staining solution; Molecular Probes, 4412S). Coverslips were rinsed with PBS, mounted, and imaged using a Leica SP8 with a 63×/1.40 oil-based objective.

### Site-directed mutagenesis

The hTRPML2(WT):YFP pcDNA3.1 construct was initially described recently used by Plesch *et al*. (*18*). Site-directed mutagenesis of hTRPML2 was performed as previously described using the QuikChange II Site-Directed Mutagenesis Kit (Agilent Technologies) according to the manufacturer’s instructions. The following primers were employed to generate mutant TRPML2 isoforms: L314R (forward: ACAAGATCCATTGTTCGTGCTCTAAGGTTACGG; reverse: CCGTAACCTTAGAGCACGAACAATGGATCTTGT) and A315G: (forward: AGATCCATTGTTCTTGGTCTAAGGTTACGGAAG; reverse: CTTCCGTAACCTTAGACCAAGAACAATGGATCT).

## Supplementary Material

http://advances.sciencemag.org/cgi/content/full/6/46/eabb5064/DC1

Movie S1

Movie S2

Movie S3

Movie S4

Movie S5

Movie S6

Adobe PDF - abb5064_SM.pdf

TRPML2 is an osmo/mechanosensitive cation channel in endolysosomal organelles

## SUPPLEMENTARY MATERIALS

Supplementary material for this article is available at http://advances.sciencemag.org/cgi/content/full/6/46/eabb5064/DC1

## Appendix

View/request a protocol for this paper from *Bio-protocol*.
