# Supplementary material for: TRPML2 is an osmo/mechanosensitive cation channel in endolysosomal organelles
Source: Sci Adv. 2020 Nov 11;6(46):eabb5064. doi: 10.1126/sciadv.abb5064 (PMC7673730; doi:10.1126/sciadv.abb5064)
Supplement: http://advances.sciencemag.org/cgi/content/full/6/46/eabb5064/DC1 [file supp_6_46_eabb5064__index.html]

Science Advances | Science AdvancesAAASSearchScience AdvancesMenu

## Supplementary Materials

# TRPML2 is an osmo/mechanosensitive cation channel in endolysosomal organelles

Cheng-Chang Chen, Einar Krogsaeter, Elisabeth S. Butz, Yanfen Li, Rosa Puertollano, Christian Wahl-Schott, Martin Biel, Christian Grimm

Download Supplement

**The PDF file includes:**

- Figs. S1 to S6
- Legends for movies S1 to S6

**Other Supplementary Material for this manuscript includes the following:**

- Movie S1
- Movie S2
- Movie S3
- Movie S4
- Movie S5
- Movie S6

**Files in this Data Supplement:**

- Adobe PDF - abb5064\_SM.pdf
- abb5064\_Movie\_S1.avi
- abb5064\_Movie\_S2.avi
- abb5064\_Movie\_S3.avi
- abb5064\_Movie\_S4.avi
- abb5064\_Movie\_S5.avi
- abb5064\_Movie\_S6.avi
