# Supplementary material for: TRPML2 is an osmo/mechanosensitive cation channel in endolysosomal organelles
Source: Sci Adv. 2020 Nov 11;6(46):eabb5064. doi: 10.1126/sciadv.abb5064 (PMC7673730; doi:10.1126/sciadv.abb5064)
Supplement: http://advances.sciencemag.org/cgi/content/full/6/46/eabb5064/DC1 [file supp_6_46_eabb5064__1.pdf]

Science Advances
AAAS

advances.sciencemag.org/cgi/content/full/6/46/eabb5064/DC1

Supplementary Materials for

TRPML2 is an osmo/mechanosensitive cation channel in endolysosomal organelles

Cheng-Chang Chen*, Einar Krogsaeter, Elisabeth S. Butz, Yanfen Li, Rosa Puertollano, Christian Wahl-Schott, Martin Biel*, Christian Grimm*

*Corresponding author. Email: cheng-chang.chen@cup.uni-muenchen.de (C.-C.C.); mbiel@cup.uni-muenchen.de (M.B.); christian.grimm@med.uni-muenchen.de (C.G.)

Published 11 November 2020, *Sci. Adv.* **6**, eabb5064 (2020)
DOI: 10.1126/sciadv.abb5064

The PDF file includes:

Figs. S1 to S6
Legends for movies S1 to S6

Other Supplementary Material for this manuscript includes the following:

(available at advances.sciencemag.org/cgi/content/full/6/46/eabb5064/DC1)

Movies S1 to S6

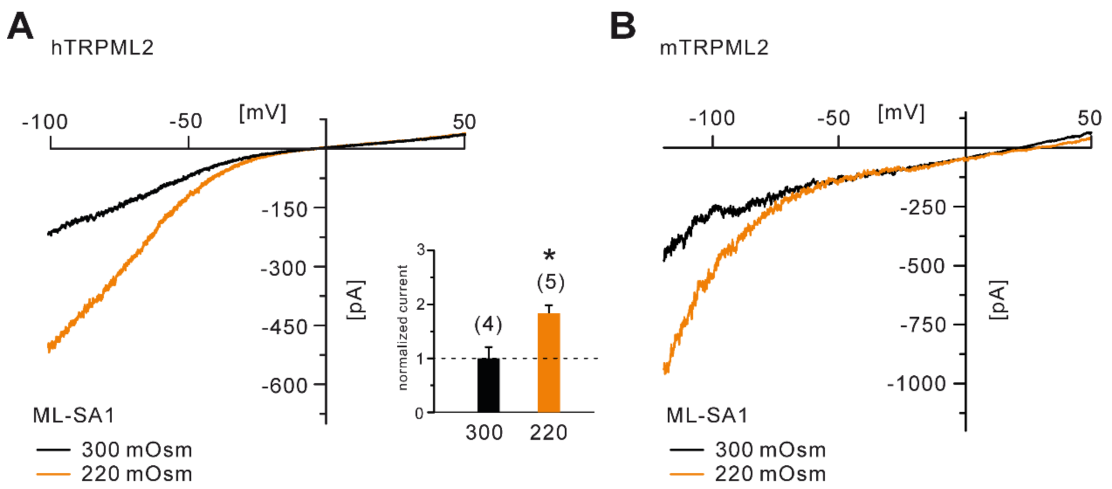

Fig. S1. Hypotonicity further increases ML-SA1 evoked TPRML2 currents. Hypotonic stimulation further increases ML-SA1 (10µM) evoked human TPRML2 currents (**A**) and mouse TRPML2 currents (**B**) in LE/LY isolated from hTRPML2- or mTRPML2-overexpressing HEK293 cells. The number of patched individual organelles is indicated in parentheses. Data are represented as mean ± SEM. * indicates $p<0.05$, Student's t-test, unpaired.

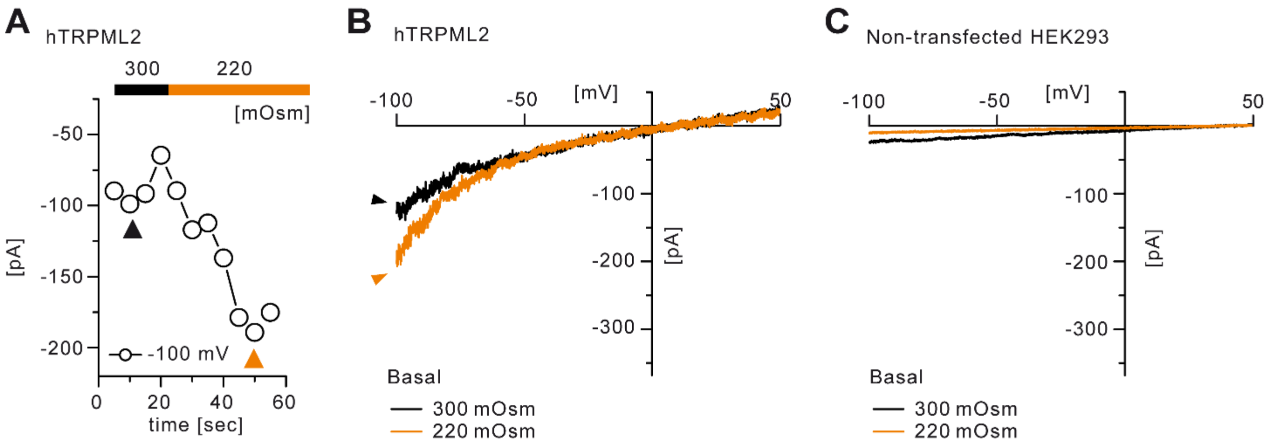

Fig. S2. Hypotonicity evoked TRPML2 currents in the absence of exogenously administered agonists. (**A**) Time-course of whole LE/LY recording (LE/LY isolated from human TRPML2-overexpressing HEK293 cells) in the absence of TRPML2 agonists using ramp protocols. Recordings at indicated time points were used for the I-V plot in (**B**). (**C**) No significant hypotonicity evoked currents were observed in LE/LY isolated from non-transfected HEK293 cells.

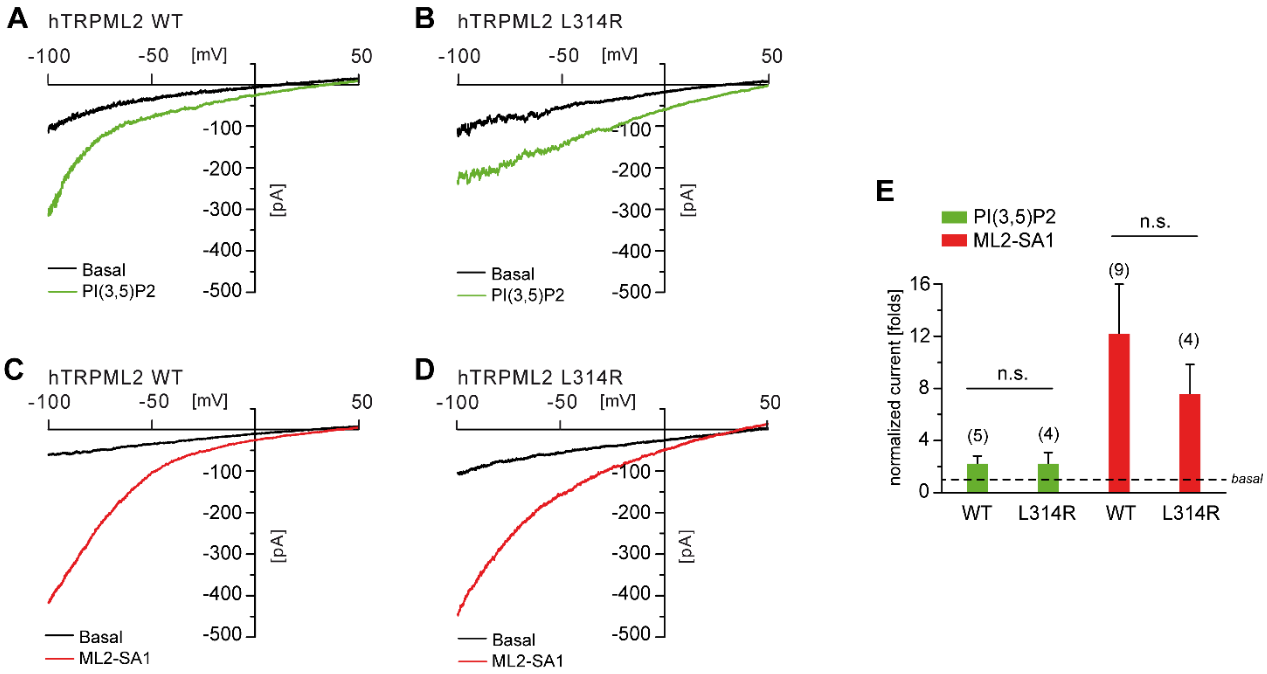

Fig. S3. Endogenous and small molecule agonist evoked TRPML2 L314R currents. 1µM $PI(3,5)P_2$ (**A-B**) and 10µM ML2-SA1 (**C-D**) evoked TRPML2 currents in LE/LY isolated from hTRPML2 WT (**A, C**) and L314R (**B, D**) overexpressing HEK293 cells. (**E**) Statistical summary of data as shown in A-D as fold increase compared to the respective current before agonist application, each. The number of patched individual organelles is indicated in parentheses. Data are represented as mean ± SEM. n.s. indicates no significant difference, Student's t-test, unpaired.

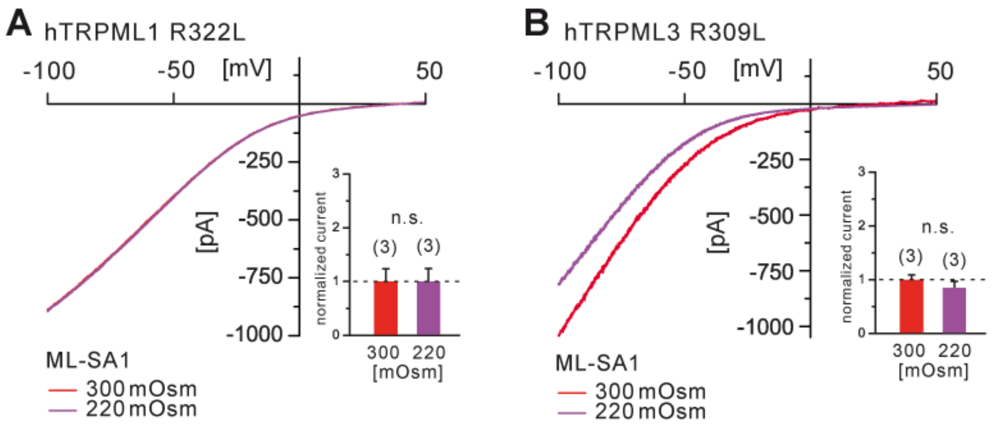

Fig. S4. Mutating TRPML1 or TRPML3 at the positions equivalent to L314 in TRPML2 (R322L and R309L). Whole-LE/LY recordings of ML-SA1 (10µM) elicited inward cation currents under isotonic or hypotonic conditions from TRPML1 R322L (**A**) and TRPML3 R309L (**B**) expressing HEK293 cells. Data are represented as mean ± SEM.

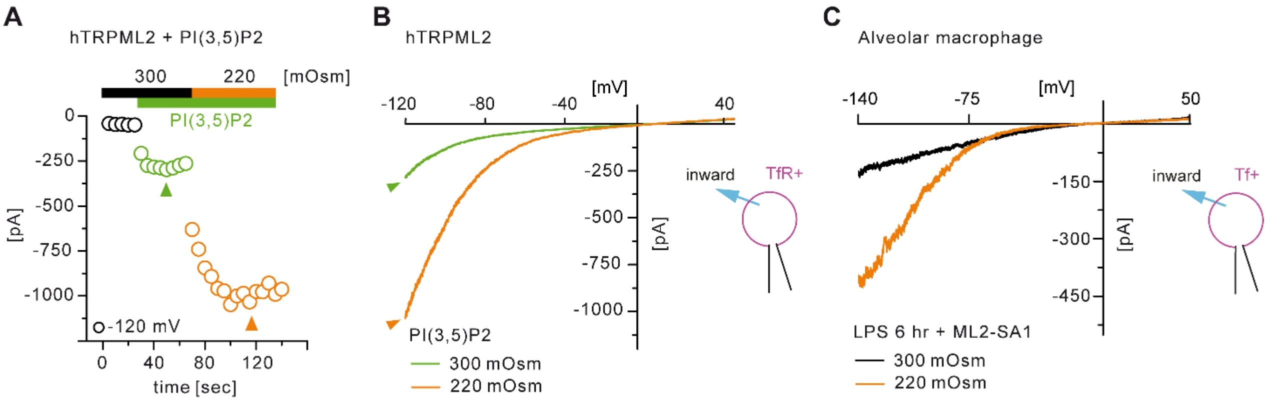

Fig. S5. Hypotonicity evoked TRPML2 currents in RE isolated from either human TRPML2 overexpressing HEK293 cells or enogenously expressing, LPS-activated macrophages. (**A**) Time-course of whole-RE (TfR+) currents elicited with PI(3,5)P_2 under isotonic versus hypotonic conditions. Recordings at the indicated time points were used for I-V relationships in (**B**). (**C**) Representative TRPML2-like currents recorded from transferrin (Tf) loaded RE isolated from LPS-stimulated alveolar macrophages after activation with ML2-SA1 in isotonic versus hypotonic solution.

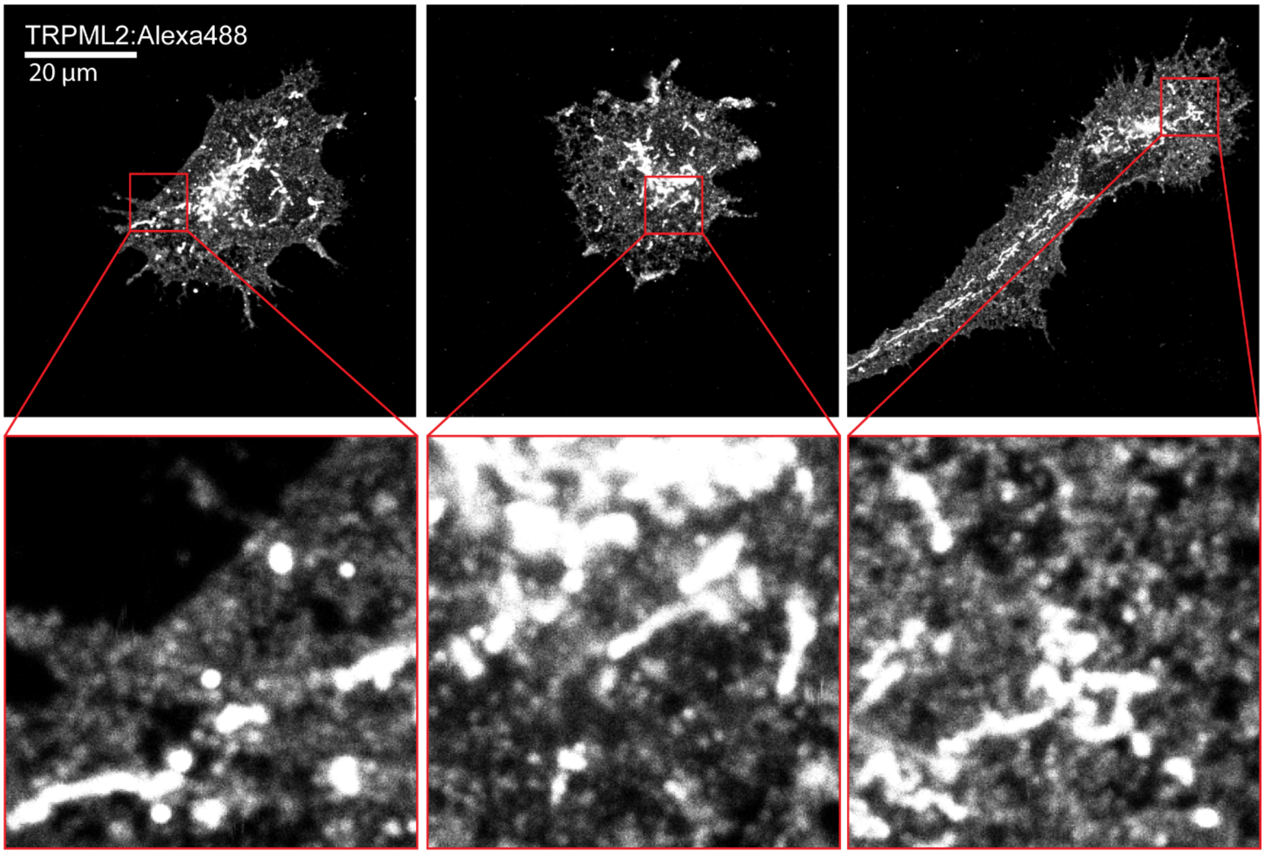

Fig. S6. Endogenously expressed TRPML2 localizes to tubular structures in LPS-stimulated bone marrow-derived macrophages. Primary murine bone marrow-derived macrophages were differentiated *in vitro* and stimulated with LPS to facilitate TRPML2 expression as previously described (*18, 21*). Macrophages were fixed and stained for TRPML2, revealing both punctate vesicular and elongated tubular staining patterns.

Movie S1. 3D rendering of transferrin-loaded WT TRPML2-expressing cell. The video shows a 360° rotation of a 8 x 8 x 19.1 µm field-of-view, indicating WT TRPML2-expressing endosomes (green) loaded with Alexa555-conjugated transferrin (red).

Movie S2. 3D rendering of transferrin-loaded L314R TRPML2-expressing cell. The video shows a 360° rotation of a 8 x 8 x 13.1 µm field-of-view, indicating L314R TRPML2-expressing endosomes (green) loaded with Alexa555-conjugated transferrin (red).

Movie S3. 3D rendering of transferrin-loaded WT TRPML2-expressing cell chased for 3 minutes. The video shows a 360° rotation of a 8 x 8 x 17.4 µm field-of-view, indicating WT TRPML2-expressing endosomes (green) loaded with Alexa555-conjugated transferrin (red), and chased for 3 minutes in the presence of 30 µM ML2-SA1 and unconjugated transferrin

Movie S4. 3D rendering of transferrin-loaded L314R TRPML2-expressing cell chased for 3 minutes. The video shows a 360° rotation of a 8 x 8 x 14.4 µm field-of-view, indicating L314R TRPML2-expressing endosomes (green) loaded with Alexa555-conjugated transferrin (red), and chased for 3 minutes in the presence of 30 µM ML2-SA1 and unconjugated transferrin

Movie S5. 3D rendering of transferrin-loaded WT TRPML2-expressing cell chased for 10 minutes. The video shows a 360° rotation of a 8 x 8 x 13.1 µm field-of-view, indicating WT TRPML2-expressing endosomes (green) loaded with Alexa555-conjugated transferrin (red), and chased for 10 minutes in the presence of 30 µM ML2-SA1 and unconjugated transferrin

Movie S6. 3D rendering of transferrin-loaded L314R TRPML2-expressing cell chased for 10 minutes. The video shows a 360° rotation of a 8 x 8 x 15.4 µm field-of-view, indicating L314R TRPML2-expressing endosomes (green) loaded with Alexa555-conjugated transferrin (red), and chased for 10 minutes in the presence of 30 µM ML2-SA1 and unconjugated transferrin.
